# Infiltration of Matrix-Non-producers Weakens the *Salmonella* Biofilm and Impairs Its Antimicrobial Tolerance and Pathogenicity

**DOI:** 10.3389/fmicb.2015.01468

**Published:** 2015-12-23

**Authors:** Chakravarthy S. Srinandan, Monalisha Elango, Divya P. Gnanadhas, Dipshikha Chakravortty

**Affiliations:** ^1^Department of Microbiology and Cell Biology, Indian Institute of ScienceBangalore, India; ^2^Biofilm Biology Lab, Centre for Research on Infectious Diseases, School of Chemical and Biotechnology, SASTRA UniversityThanjavur, India; ^3^Department of Aerospace Engineering, Indian Institute of ScienceBangalore, India; ^4^Centre for Biosystems Science and Engineering, Indian Institute of ScienceBangalore, India

**Keywords:** *Salmonella*, biofilm, public good, cooperation, spatial structure

## Abstract

Bacterial biofilms display a collective lifestyle, wherein the cells secrete extracellular polymeric substances (EPS) that helps in adhesion, aggregation, stability, and to protect the bacteria from antimicrobials. We asked whether the EPS could act as a public good for the biofilm and observed that infiltration of cells that do not produce matrix components weakened the biofilm of *Salmonella enterica* serovar Typhimurium. EPS production was costly for the producing cells, as indicated by a significant reduction in the fitness of wild type (WT) cells during competitive planktonic growth relative to the non-producers. Infiltration frequency of non-producers in the biofilm showed a concomitant decrease in overall productivity. It was apparent in the confocal images that the non-producing cells benefit from the EPS produced by the Wild Type (WT) to stay in the biofilm. The biofilm containing non-producing cells were more significantly susceptible to sodium hypochlorite and ciprofloxacin treatment than the WT biofilm. Biofilm infiltrated with non-producers delayed the pathogenesis, as tested in a murine model. The cell types were spatially assorted, with non-producers being edged out in the biofilm. However, cellulose was found to act as a barrier to keep the non-producers away from the WT microcolony. Our results show that the infiltration of non-cooperating cell types can substantially weaken the biofilm making it vulnerable to antibacterials and delay their pathogenesis. Cellulose, a component of EPS, was shown to play a pivotal role of acting as the main public good, and to edge-out the non-producers away from the cooperating microcolony.

## Introduction

Salmonellosis is the leading cause of food borne diseases worldwide (Kothari et al., 2008; Westrell et al., 2009; CDC, 2011). In addition to the virulence factors contributing to success of acute infections, *Salmonella* uses its ability to form biofilm lifestyle to survive under stress conditions both inside as well as outside the host. *Salmonella* biofilms are known to occur on different surfaces including water distribution systems, food processing equipment, plant, and epithelial surfaces, while they also form persistent biofilms on gall stones in the host (Steenackers et al., 2012). Cells in a biofilm are notorious for their tolerance toward high doses of antimicrobials relative to their planktonic counterparts. Apart from known mechanisms of antimicrobial resistance, EPS in the biofilm limits the penetration of antibiotics along with the differential physiological activities in the biofilm population that may provide them additional protection. *Salmonella* biofilm cells are resilient to high doses of chlorine and other sanitizers, especially due to the EPS matrix, and they pose potential risks in water distribution systems and food processing units (Joseph et al., 2001; Solano et al., 2002; Scher et al., 2005; Corcoran et al., 2014).

Biofilms are cell collectives of bacteria embedded in an extracellular matrix. The three-dimensional biofilm architecture presents a spatially heterogeneous structure in terms of nutrient gradients, metabolites and, physiology of the cells (Stewart and Franklin, 2008). This architecture is mainly a function of the EPS produced by the biofilm cells (Yang et al., 2011; Berk et al., 2012). The EPS components of *Salmonella* predominantly consist of cellulose, curli, and BapA adhesin. These EPS components act as a scaffold to the cells, providing them structural stability (Römling et al., 1998a; Zogaj et al., 2001; Solano et al., 2002; Latasa et al., 2005). Production of EPS is controlled by CsgD, which is a 216 amino acids long transcriptional regulator of the LuxR family, and its expression is influenced by several environmental factors including oxygen and temperature (Römling et al., 1998b; Gerstel and Römling, 2001).

Biofilm cells are likely to cooperate by secreting certain potential public goods like the siderophores, chitinases, proteases, surfactants, etc., which are produced by an individual that can be utilized by the producer and its neighbors (West et al., 2007; Nadell et al., 2009; Drescher et al., 2014). Gestel et al. (2014) showed that the EPS produced by *Bacillus subtilis* could act as a public good by facilitating spreading. The EPS matrix confers several other benefits to the bacterial cells like adhesion, aggregation, retention of water, resilience to antimicrobials, etc., (Flemming and Wingender, 2010). However, critical to the cooperators is the emergence of non-producers that do not contribute to the public good production, but exploit it. The emergence of these free riders that do not pay the fitness cost could lead to a decline of the cooperative system (Rainey and Rainey, 2003). Nevertheless, a cooperative system can evolve various strategies to deter conflicts, like the limited dispersal or kin discrimination (Travisano and Velicer, 2004). For example, spatial segregation of the producers and non-producers can help in maintenance of cooperation (Gestel et al., 2014). Drescher et al. (2014) observed that the EPS in biofilms could help solve the secreted public good (chitinase, in this case) dilemma. In this study, we asked whether the EPS matrix produced by *Salmonella* acts as a public good in the biofilm context. Moreover, as *Salmonella* biofilms are importantly implicated in medicine, we looked at different consequences of the infiltration of EPS-non-producing cells on the antibacterial properties and infection capability of the biofilms. Spatial structure was also looked at, to understand the arrangement of these cell types in the biofilms, which led us to know other roles of cellulose in the biofilm.

## Materials and methods

### Bacterial strains and culture conditions

*Salmonella enterica* serovar Typhimurium 14028 strain was used for all the experiments. The *csgD* and *bcsA* genes were inactivated according to Datsenko and Wanner (2000) method with the primer sequences given in the Table S1. The pFPV 25.1 plasmid containing GFPmut3 gene (Valdivia and Falkow, 1996) and pFPVmcherry/2 (Drecktrah et al., 2008) were used to transform the cells for confocal microscopic experiments. LB medium without salt and incubation at room temperature (25–28°C) was used to culture bacteria for all biofilm experiments. All the chemicals including antibiotics used in this study were purchased from HiMedia Labs, India.

### Competition experiments and fitness determination

The *in vitro* competition experiment between WT and Δ*csgD* planktonic cells were done by co-culturing and incubating them in low shaking conditions of 60 rpm and maintained at 27°C (henceforth called as LS) and high shaking conditions of 176 rpm, maintained at 37°C (henceforth called as HS). Both the strains were grown overnight individually in LB media, which was centrifuged and washed twice in sterile phosphate buffered saline (PBS). The culture was independently inoculated into a fresh LB medium and the absorbance of the log phase grown culture was adjusted to 0.1 with LB in a spectrophotometer. Both the cultures were inoculated in a fresh LB tube with 1:1 ratio, incubated and plated at 5 and 96 h for analyzing their fitness at log and stationary phase, respectively. Colony forming units (CFU) were enumerated by plating them on LB, with and without chloramphenicol plates. Absolute fitness was estimated by Malthusian parameter (*M*) according to Lenski et al. (1991) with *M* = *ln(N*_1_*/N*_0_*)*, where *N*_0_ is the initial cell count at 0 h, *N*_1_ is the final viable cell count at 5 or 96 h for log and stationary phase, respectively. Relative fitness is the *M*_Δ__*csgD*_*/M*_*WT*_.

### Quantitative PCR for gene expression

Assessment of the expression of *csgD* was carried out using qPCR. Briefly, RNA was isolated from *Salmonella* Typhimurium grown at different growth conditions of LS and HS for 96 h (stationary phase) of growth using TRIzol (Life Technologies) as per manufacturer's protocol and reverse transcribed using random hexamers (NEB) and Tetro reverse transcriptase (Bioline) as per standard protocol. The cDNA was diluted and analyzed for the presence of *csgD* using specific primers given in Table S1 by qPCR SYBR® FAST Master Mix (Kapa Biosystems) in an Applied Biosystems® ViiA™ 7 Real time PCR instrument. Expression was normalized to the housekeeping 16S rRNA gene.

### Biofilm experiments and antimicrobial sensitivity

The submerged biofilm experiment was performed in 24-well microtitre plates. Two milliliters of LB media was dispensed in the wells and inoculated with around 10^7^ cells of an overnight culture. The microtitre plates were incubated under static condition for 3 days, the wells were washed thrice with PBS and the biofilm was stained with 1% crystal violet dye solution for 15 min, and again the wells were rinsed thrice with PBS to wash off the unbound dye. Quantification of the biofilm biomass was done by de-staining the crystal violet with methanol and recording the absorbance according to Srinandan et al. (2010). Similar procedure as the submerged biofilm was performed in static incubation at room temperature for visualization of biofilm pellicle in 24-well microtitre plates. The pellicle was removed for further analysis with the help of tweezers or directly removed using glass coverslips (1 cm in diameter) to which it adheres. Dry weight of the pellicle was determined by removing the pellicle with a pre-weighed glass coverslips, drying them in hot air oven for 1 h at 70°C. For antimicrobial sensitivity experiments, the biofilm was removed carefully with tweezers and exposed to ciprofloxacin (4 μg/ml) or sodium hypochlorite (at varying concentrations up to 400 ppm), for 1 h in shaking condition (176 rpm at 37°C). Ciprofloxacin and sodium hypochlorite were removed and the biofilm was disrupted by glass beads of size 0.5 mm diameter in a Mini bead beater (BioSpec) with maximum speed. The cultures were plated in respective antibiotic plates for the enumeration of CFU.

### Scanning electron microscopy (SEM)

The developing biofilm in the microtitre plate wells were carefully taken in a sterile glass coverslip. The samples were fixed in 2.5% glutaraldehyde at 4°C for 24 h. After washing with PBS for three times, the samples were dehydrated in a gradient series of alcohol concentration (50, 70, 80, 90, and 100%) for 10 min at each concentration. The samples were sputter-coated with gold (JEOL JFC-1100E ion sputtering device; JEOL, Tokyo, Japan) and analyzed by field emission-SEM (FEI Sirion, Eindhoven, The Netherlands).

### Confocal microscopy and image analysis

Image acquisition of the biofilm was done in Zeiss confocal microscope (LSM Meta 710). More than 10 random fields per sample were captured with a 20X objective. Confocal settings were nearly similar for each experimental set-up. Thresholding of the confocal images was carried out to remove the noise and surfaces were added in the Surpass scene viewer of the Imaris software (Bitplane Inc.). ADOBE Photoshop® 7.0 (Adobe systems Inc) was used for routine processing of the images. Spatial arrangement was analyzed in the DAIME software (Daims et al., 2006), where the images were first segmented automatically in 2-D stacks. Stereological analyses was then carried out by two population analyses by using the linear dipole method to quantify pair cross-correlation *g(r)*, between the cell types. The values of *g(r)* determine the positive or negative correlation of the cells types at different distances *(r)*. If both cell types in the biofilm cluster together at distance *r, g(r)* will be greater than 1.0, whereas *g(r)* will be lower than 1.0 if the cells are away from each other at distance *r*. Random distribution of the cell types is envisaged if the *g(r)* is equal to 1.0 (Daims et al., 2006). COMSTAT program, a script written in MATLAB (Heydorn et al., 2000) was used to quantify the biofilm parameters like the biovolume and thickness.

### *In vivo* experiments

All the experiments were carried out in accordance with the approved guidelines of Institutional Animal Ethics Committee at Indian Institute of Science (IISc), Bangalore, India (Registration No: 48/1999/CPCSEA) and National Animal Care Guidelines were strictly followed. Six to eight weeks old BALB/c mice were bred and housed at the Central Animal Facility, IISc, and used for all the experiments. The biofilm was removed from the wells of microtitre plates with sterile tweezers, disrupted mildly by glass beads of size 0.5 mm diameter with very low speed in a Mini bead beater (BioSpec), and infected orally to BALB/c mice. The infectious dose was nearly 10^7^ cells ml^−1^ when they were administered individually, whereas around 5 × 10^6^ cells ml^−1^ of each cell type was infected when co-cultured. The mice were sacrificed and the mesenteric lymph node (MLN), spleen, and liver were aseptically isolated, homogenized, and plated in respective antibiotic plates. The relative pathogenesis index (RPI) was calculated as *RPI* = *PB*_*co*−*culture*_*/PB*_*WT*_, where PB = Pathogenic burden normalized to its input inoculum. *RPI* value will tell the capability of the non-producer infiltrated and the WT biofilm cells to cause the pathogenesis. Competitive index (*CI*) was calculated according to Stecher et al. (2008), where *CI* = *Ratio*_*output*_*/Ratio*_*input*_. CI value will give the insight into the capacity of the cell types to infect different organs.

### Statistical data analysis

All the data were analyzed in the GraphPad Prism software. One-sample *t*-test with a theoretical mean of 1.0 was used to determine significance for independent data sets. Two-tailed unpaired *t*-test or non-parametric Mann-Whitney *U* test was used to compare two experimental groups. One-way ANOVA with Bonferroni *post-hoc* test was used to determine significance between multiple experimental groups. *P* < 0.05 was considered significant.

## Results

### Biofilm matrix production is metabolically costly

**Figure 1:**
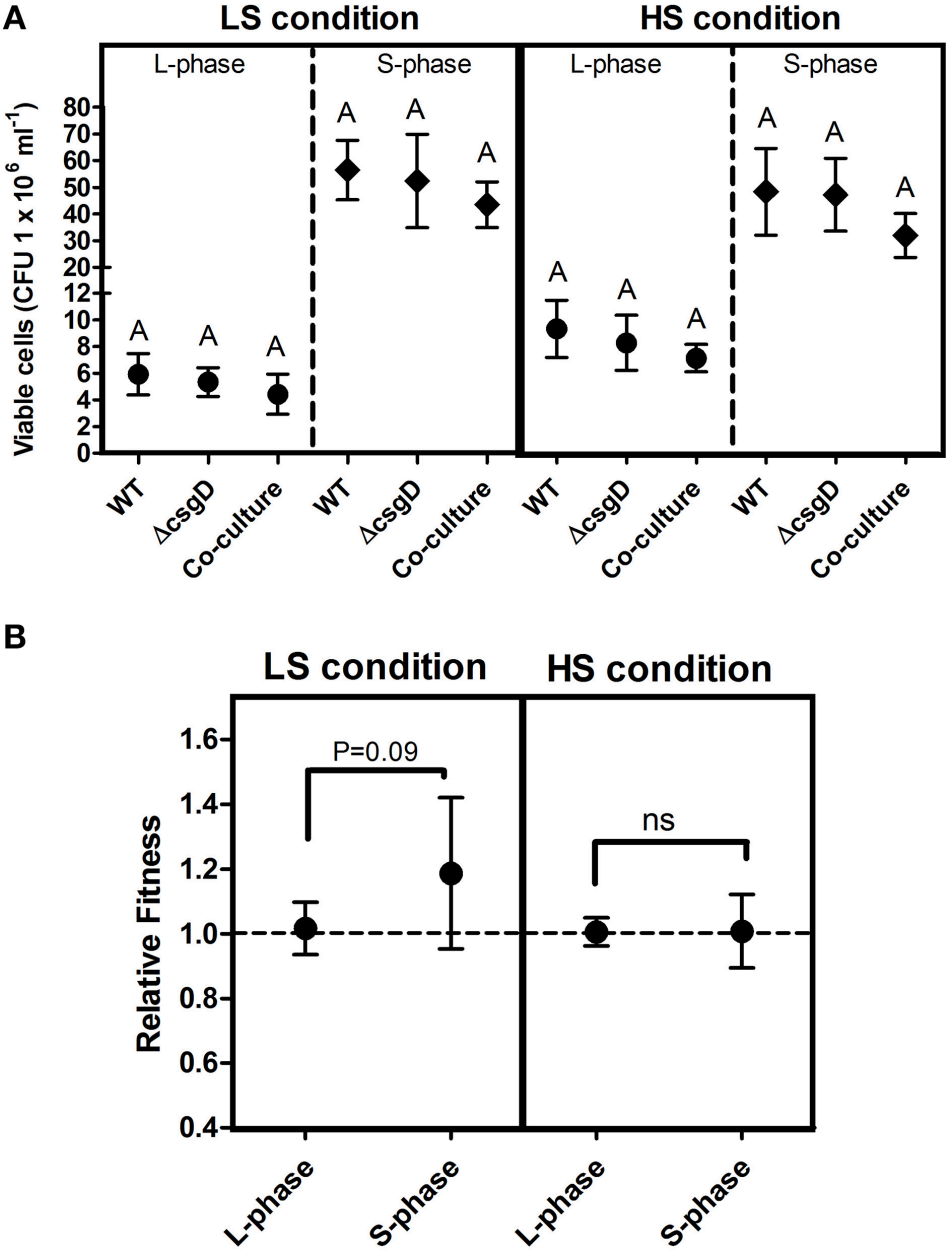
**Planktonic growth analysis in mono- and co-cultures**. **(A)** Growth (cfu ml^−1^) of WT and the Δ*csgD* cells at LS condition (incubation temperature was maintained at 27°C with low shaking of 60 rpm) and the HS condition (incubation temperature was maintained at 37°C with high shaking at 176 rpm). **(B)** Relative fitness as Malthusian growth parameter of WT and Δ*csgD* in the competition experiment, estimated at 5 and 96 h of growth to assess their competitive ability. Error bars indicate 95% CI of the mean (*n* ≥ 5). One-way ANOVA with Bonferroni *post-hoc* test was performed to analyze the data in **(A)**, and one-sample *t*-test was performed on **(B)** to determine significance. Same letters on the data point indicate that they are not significant (ns = not significant).

The EPS matrix provides structural support and shields the biofilm cells from antimicrobials, apart from their other known functions. The major components of EPS for biofilm formation is activated by *csgD* gene. We reasoned that the Wild Type bacteria (WT), which form biofilm would incur a metabolic cost to produce EPS. The cultures were grown in monocultures and a co-culture of WT and Δ*csgD* cells were grown in 1:1 ratio, and the colony forming units (cfu) was measured at log and stationary phases. Substantial difference was not observed in the planktonic growth productivity in either the mono- or co-culture (Figure 1A). The Malthusian growth parameter of both WT and the Δ*csgD* cells were measured from the co-culture grown cultures under both HS and LS conditions. At LS condition, Malthusian fitness of co-culture grown Δ*csgD* cells was significantly (Mann-Whitney *U* test, *P* < 0.01, *n* ≥ 5) higher in the stationary phase. However, no substantial differences were observed in the fitness between the cell types in both the logarithmic and stationary phase at HS condition. But the relative fitness of Δ*csgD* cells was marginally significant (one sample *t*-test, *P* = 0.09, *n* ≥ 5) at stationary phase of LS condition for Δ*csgD* cells in stationary phase (Figure 1B). Expression of the *csgD* gene was monitored at both conditions in stationary phase by qPCR, which showed a 6.6-fold higher expression levels at LS condition than HS (Figure S1). The LS condition induced the WT cells to produce EPS, thus incurring metabolic cost and leading to a lower fitness than the Δ*csgD* cells.

### Biofilm matrix as a public good

**Figure 2:**
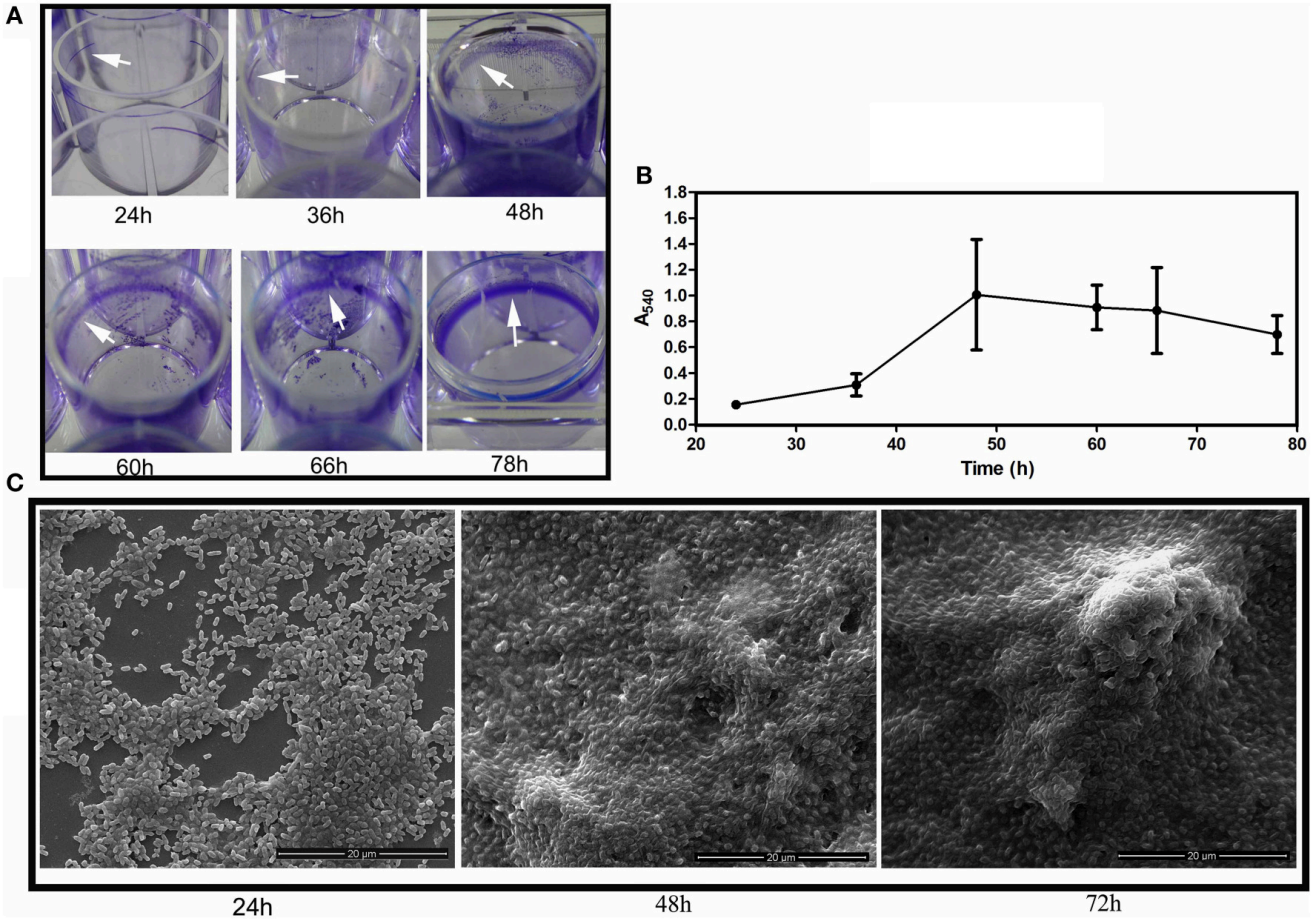
**Biofilm development**. **(A)** Development of WT submerged biofilm on the plastic surface of microtitre wells. White arrowheads point toward the biofilm ring formed on the plastic surfaces. **(B)** Quantification of the biofilm biomass at different time intervals by the crystal violet de-staining method. Error bars indicate SEM (*n* ≥ 6) and **(C)** Scanning Electron Microscopic images depicting the pellicle development at 24, 48, and 72 h.

Most of the biofilm-related studies in literature can be found on solid/liquid or the solid/liquid/air interfacial biofilm, where the cells are adhered to the substratum surface (henceforth called as submerged biofilm). When incubated for 3 days in room temperature, under static conditions in the LB medium, *S*. Typhimurium forms biofilm pellicle at the air and liquid interface as well as the submerged biofilm can be observed on the edges of the substratum. It is thought that the submerged biofilm precedes the pellicle formation and might possibly provide the attachment sites for the pellicle stability (Scher et al., 2005). However, both kinds of biofilm developed nearly simultaneously, which could be visualized from 24 h of incubation (Figure 2). Submerged biofilm was seen at the substratum surface as a ring (Figure 2A), while clusters of bacteria could be seen at 24 h in the air-liquid interface when visualized under the electron microscope (Figure 2C).

**Figure 3:**
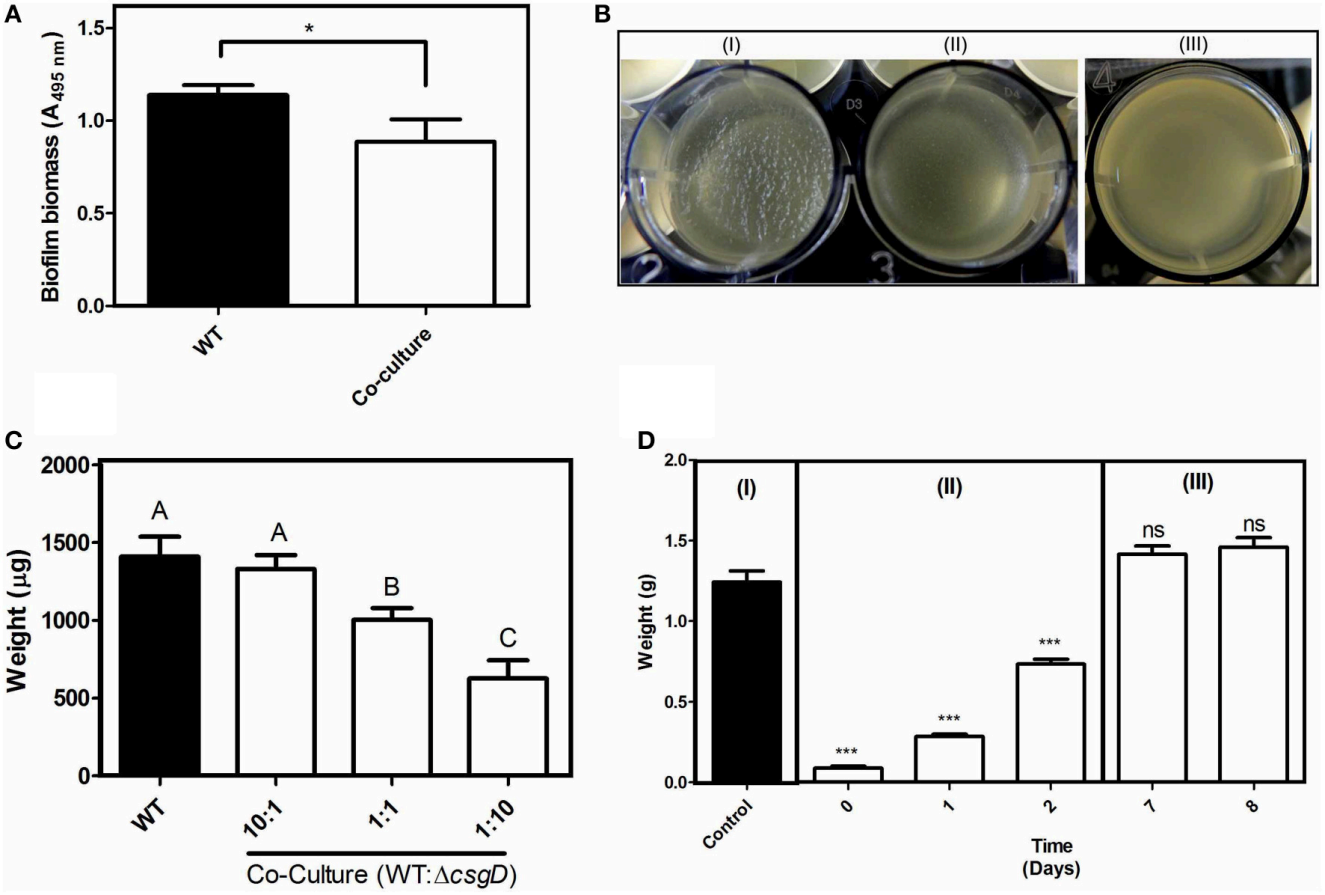
**Consequence of infiltration of non-producers in the biofilm**. **(A)** Biomass of submerged biofilm, as estimated by the crystal violet destaining method. The inoculum for the co-cultured biofilm consisted of a 1:1 ratio of WT and Δ*csgD*. **(B)** Biofilm Pellicle of (I) WT, (II) Co-culture of WT + Δ*csgD* in 1:1 ratio, and (III) Only Δ*csgD*. **(C)** Dry weight of the biofilm pellicle formed in different ratios of WT + Δ*csgD*. **(D)** Glass bead assay to determine strength of the pellicle (I) 3rd day biofilm of WT is the control (II) Δ*csgD* cells were added to the culture broth on different days during the biofilm formation and glass bead assay was done to determine the strength on 3rd day biofilm (III) Δ*csgD* cells were added on 3rd day old biofilm and the assay was done on 7th and 8th day old biofilm. Error bars indicate the SEM (*n* ≥ 6). Unpaired *t*-test was performed to determine the significance in relation to the control biofilm in **(A,D)**. One-way ANOVA with Bonferroni *post-hoc* test was used to determine the significance for **(C)**. Same letters on the data point indicate that they are not significant (^***^P < 0.001, ^*^P < 0.05, ns = not significant).

Cells in the biofilm pellicle benefit from oxygen accessibility while they restrict the oxygen access to cells unable to form biofilm (Rainey and Rainey, 2003). The EPS components provide structural support to cells in the biofilm, and biofilm forming bacteria pay the cost to produce the matrix (Figure 1). However, the EPS-non-producers reap the benefits of staying in a biofilm without contributing to production of the matrix (Rainey and Rainey, 2003). The EPS can act as public good in the biofilm and the matrix-producing phenotype may become costly for the producers in the presence of non-producers. Therefore, if non-producing cells are introduced, they should theoretically destroy or substantially weaken the biofilm system. With the above hypothesis, we co-cultured WT and Δ*csgD* (non-producing cells) in a 1:1 ratio and incubated them for the formation of biofilm. A substantial reduction in the submerged biofilm biomass (Unpaired *t*-test, *P* = 0.039, *n* ≥ 6) was observed (Figure 3A). The co-culture also weakened the formation of biofilm pellicle substantially (Figure 3B). The WT formed wrinkly pellicle, but the co-cultured pellicle was smooth and fragile with no apparent wrinkle formation (Figure 3B and Video S1). Productivity, as tested by dry weight was reduced in the co-cultured biofilm pellicle at similar and higher frequencies of non-producers (Figure 3C). The WT pellicle could withstand a weight of more than 1.2 grams of glass beads (0.5 mm diameter in size). However, pellicle strength was tested after addition of the non-producers, during and post-biofilm formation. It was observed that the time of addition of non-producing cells played an important role in the strength of pellicle with early addition resulting in a weaker pellicle. However, addition of non-producing cells post-biofilm formation had no effect on its strength (Figure 3D). Nevertheless, presence of non-producers during biofilm development weakened the cohesiveness of the pellicle and also significantly reduced the productivity, as measured by dry weight (One-way ANOVA, *P* < 0.001, *n* ≥ 5).

### Infiltration of non-producers reduces biofilm tolerance to antimicrobials

*Salmonella* cells in biofilm are known to be tolerant to higher concentrations of chlorine (Joseph et al., 2001; Solano et al., 2002; Scher et al., 2005). *S*. Typhimurium biofilm was shown to be 2000-fold tolerant to ciprofloxacin antibiotic relative to their planktonic counterparts (Tabak et al., 2009). We speculated that the non-producer population, which reduced the rigidity (Figure 3), could possibly sensitize the biofilm cells toward antimicrobial compounds. *Salmonella* strains having the minimum inhibitory concentration (MIC) of < 1 μg ml^−1^ toward ciprofloxacin are categorized as susceptible strains, and the MIC of *S*. Typhimurium 14028 is ~0.02 μg ml^−1^ (Lee et al., 2009; Gnanadhas et al., 2013).

**Figure 4:**
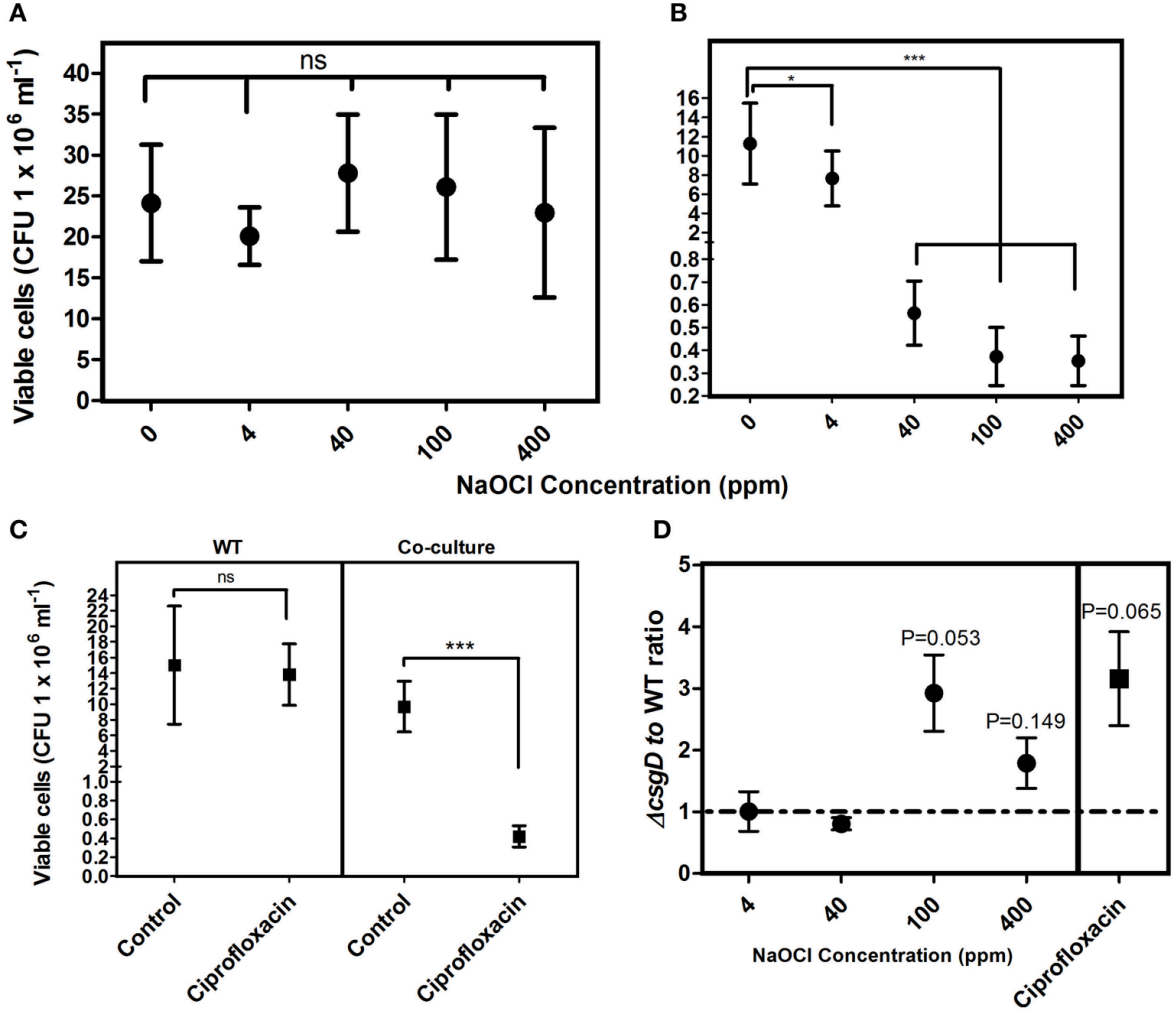
**Sensitivity of the biofilm toward NaClO and ciprofloxacin (4 μg ml^−1^). (A)** Susceptibility of the WT biofilm cells to NaClO at varying concentrations and **(B)** Susceptibility of co-cultured biofilm cells to NaClO **(C**) Sensitivity of the biofilm cells of WT and co-culture to ciprofloxacin. **(D)** Antimicrobial susceptibility ratio of the WT and Δ*csgD* cell types in the co-cultured biofilm. Error bars indicate 95% CI of the mean (*n* ≥ 4). Unpaired *t*-test was used to determine significance for **(A–C)** relative to the control. One sample *t*-test was used to determine the significance in **(D)**. (^*^P < 0.05, ^***^P < 0.001, ns = not significant).

Sensitivity of the biofilm to various concentrations of sodium hypochlorite, and 4 μg ml^−1^ ciprofloxacin was checked. The WT biofilm was tolerant to the treatment of both sodium hypochlorite and ciprofloxacin (Figure 4); however, the biofilm infiltrated with the non-producers showed increased sensitivity to these antibacterials (Figure 4B). There was a 1.5-fold reduction in the viable cells with 4 ppm, and more than a 20-fold reduction in viable cells with increasing sodium hypochlorite concentrations relative to the control co-cultured biofilm. With ciprofloxacin treatment, more than 20-fold reduction in the number of viable cells relative to the control co-cultured biofilm was observed (Figure 4C). However, the non-producers were more susceptible to hypochlorite at its higher concentrations, while they showed 3.1-folds susceptibility to ciprofloxacin than the WT cells (Figure 4D).

### Infiltration of non-producers reduces pathogenesis during initial stages

**Figure 5:**
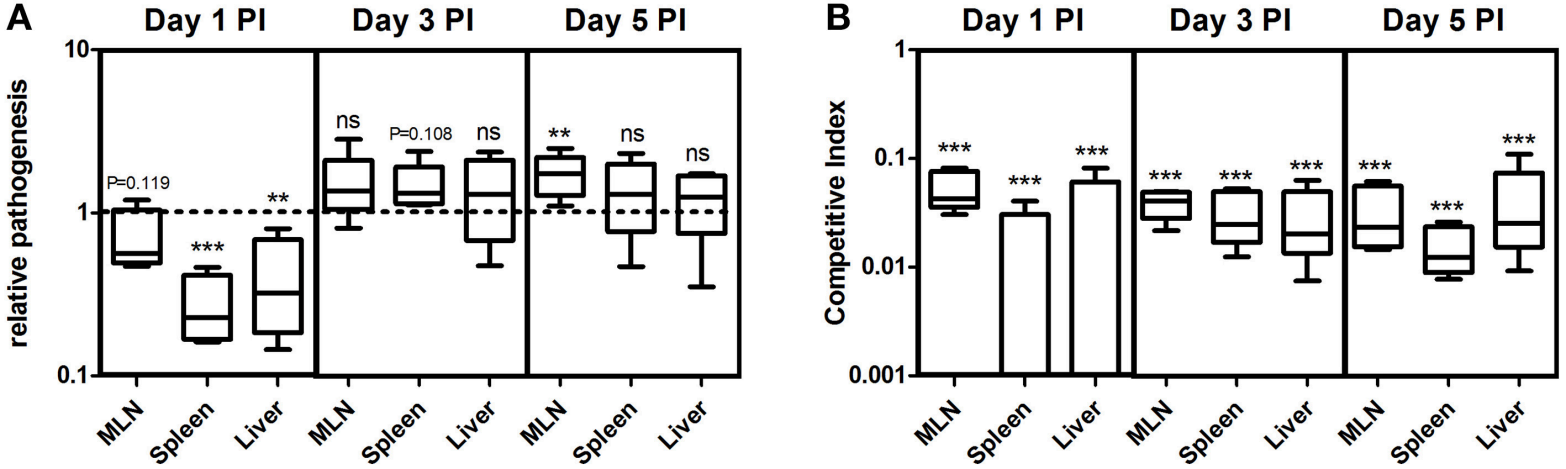
**(A)** Relative pathogenesis index (RPI) of non-producers-infiltrated biofilm relative to WT biofilm in different organs of the mice on day 1, 3, and 5 post-infection (PI). **(B)** Competitive index of Δ*csgD* relative to WT in the mice infected with co-cultured biofilm. Error bars indicate the SEM (*n* ≥ 5). One sample *t*-test was used to determine the significance. (^**^P < 0.01,^***^P < 0.001, ns ≥ not significant).

It is more likely that the *Salmonella* infections occur from a biofilm source, as bacteria predominantly survives in this lifestyle in natural settings (Thomas and McMeekin, 1981; Fett, 2000; Kroupitski et al., 2009). However, non-biofilm forming variants are commonly found in nature. For example, Solano et al. (2002) reported different frequencies of biofilm formers from isolates of *Salmonella enterica* from various sources. In addition, there are other reports showing *Salmonella* strains unable to form biofilm (Stepanovic et al., 2004; Turki et al., 2012). But, there are no reports to our knowledge on the capacity of non-biofilm-former infiltrated biofilm to infect hosts. Therefore, we performed experiments in murine salmonellosis model to measure the pathogenic potential of non-producer-infiltrated biofilm. We first measured the relative pathogenic burden of the cells from the WT biofilm and co-cultured biofilm in different organs of the mice by normalizing the CFU per organ by the initial infectious dose, which was called as the relative pathogenesis index (RPI). It was interesting to observe that the RPI was less for the co-cultured biofilm cells in all the three organs on day-1 post-infection (PI) (Figure 5A). The infection was low in MLN (one sample *t*-test, *P* = 0.119, *n* = 5), whereas highly significant in spleen (one sample *t*-test, *P* = 0.001, *n* = 5) and liver (one sample *t*-test, *P* = 0.023, *n* = 5) on day 1 PI. However, on the 3rd and 5th day PI it was higher than 1.0 (Figure 5A) indicating a delayed pathogenesis. The competitive index (CI) was estimated between the WT and non-producers from the co-cultured biofilm infection and it was observed that the CI was less for the non-producer cells (one sample *t*-test, *P* ≤ 0.001, *n* = 5) on all days and in all the organs, suggesting the importance of biofilm formation during pathogenesis (Figure 5B). The infection with individual cell types of planktonic cells showed that pathogenesis is significantly (Mann-Whitney *U* test, *P* < 0.01, *n* = 5) reduced in non-producing cell type than WT (Figure S2). Altogether, these results suggest that the non-producer-infiltrated biofilm has a reduced capacity during initial stages of pathogenesis and the *csgD*-dependent matrix may play an important role during virulence.

### Cell types are spatially assorted in the biofilm

**Figure 6:**
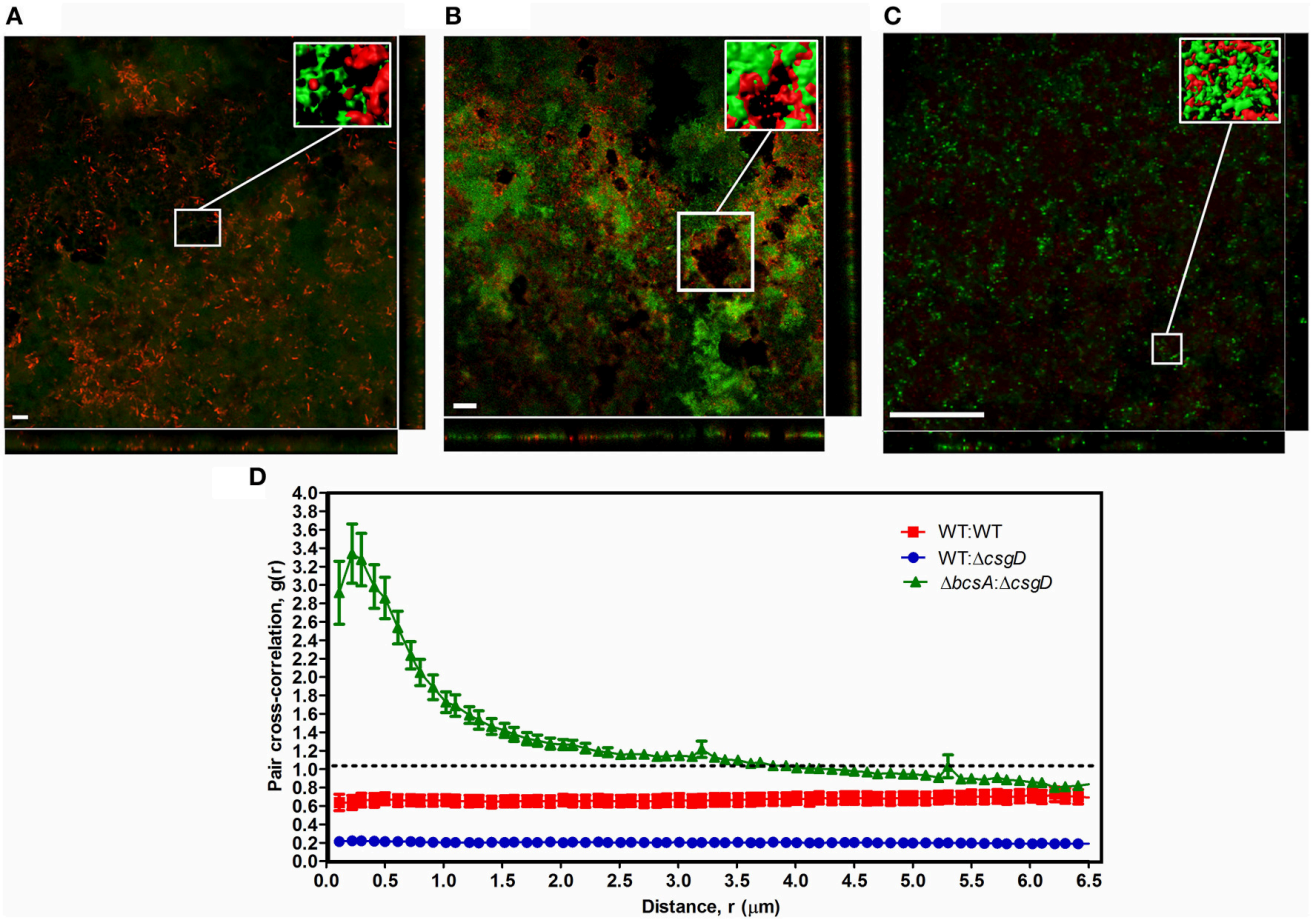
**Spatial structure of cell types in the biofilm pellicle with representative confocal microscopic image**. **(A)** WT cells expressing GFP (green) and WT cells expressing mcherry/2 (red), **(B)** WT cells expressing GFP (green) and Δ*csgD* expressing mcherry/2 (red), and **(C)** Δ*csgD* expressing GFP (green) and Δ*bcsA* expressing mcherry/2 (red). Inset is the processed image with reconstructed 3-D surfaces of the region shown in the box. **(D)** Spatial arrangement of the WT and Δ*csgD* cells with pair cross-correlation values at different distances up to 6 μm. Linear dipole method was used to determine pair cross-correlation *g(r)* values.If both cell types cluster together at distance *r, g(r)* will be greater than 1.0, whereas *g(r)* will be lower than 1.0 if the cells stay away from each other at distance *r*.(*n* ≥ 11).

**Figure 7:**
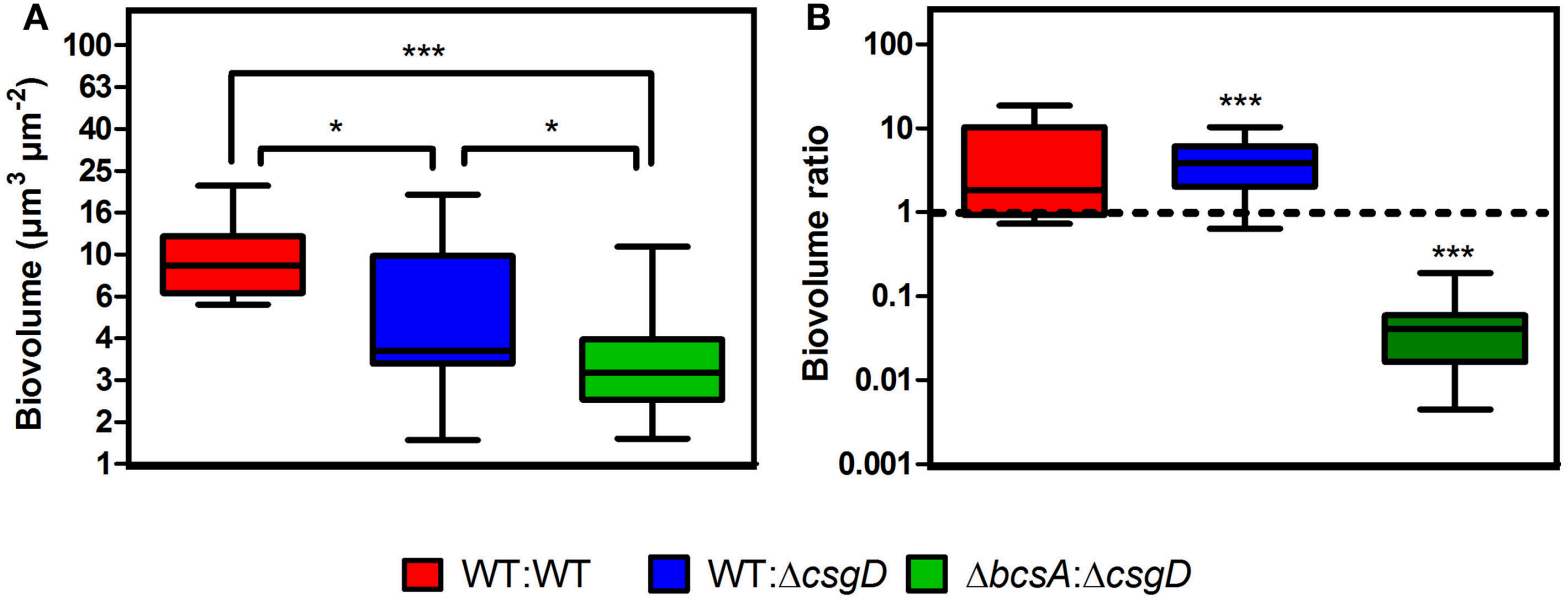
**Quantification results of the biofilm confocal images used in Figure 6**. **(A)** Biovolume and **(B)** Biovolume ratio. Error bars indicate SEM (n ≥ 11). Mann-Whitney *U* test was performed to analyze the data relative to the WT in **(A)**. One sample *t*-test was used to determine the significance in **(B)**. (^*^P < 0.05, ^***^P < 0.001).

Furthermore, the spatial arrangement of WT and non-producing cell types in the pellicle was analyzed and for this, the *S*. Typhimurium cells fluorescing green (GFP) and red (mcherry/2) were used. The pellicles became flat when they were isolated on the coverslip with the tweezers, however, no substantial differences between WT and the co-cultured pellicles in terms of thickness was observed. An average thickness of 19.26 ± 10.39 and 16.75 ± 4.68 μm, and a maximum thickness of 26.54 ± 10.46 and 30.16 ± 10.19 μm were observed respectively for WT biofilm and 1:1 ratio co-cultured biofilm. From the confocal image, it is apparent that the non-producers are at the periphery of WT microcolony in the co-cultured biofilm, while distribution of the cell types in WT biofilm is nearly random (Figures 6A,B). A significant (Mann-Whitney *U* test, *P* = 0.025, *n* ≥ 11) reduction in the biovolume between the WT and co-culture biofilm was observed (Figure 7). The biovolume ratio of WT to Δ*csgD* cells was around 4.0 and the WT (GFP) to WT (mcherry/2) was above 1.0 (Figure 7). However, spatial structure analysis revealed a negative pair cross-correlation value in the range 0.20 ± 0.007 for the co-cultured biofilm of WT and Δ*csgD* up to 6 μm distances (Figure 6D). The paired cross-correlation value for the biofilm co-cultured with a 1:1 ratio of WT (GFP) and WT (mcherry/2) was 0.67 ± 0.025 on the 3-day-old biofilm (Figure 6D). Altogether, these results suggested that the WT and non-producing cell types were spatially assorted in the biofilm.

### Cellulose keeps the non-producers at bay

**Figure 8:**
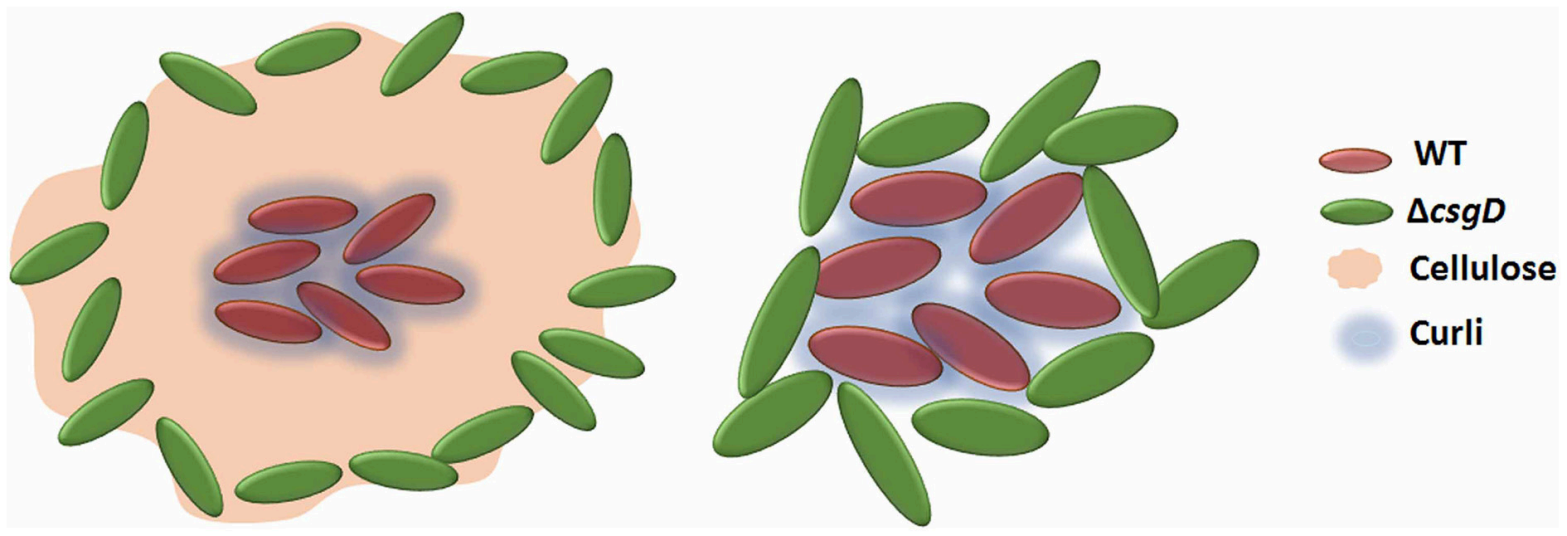
**Schematic showing that the presence of cellulose in WT biofilm keeps the non-producers away from its microcolony and in its presence, curli proteins may act as a club good for the matrix producers**.

**Figure 9:**
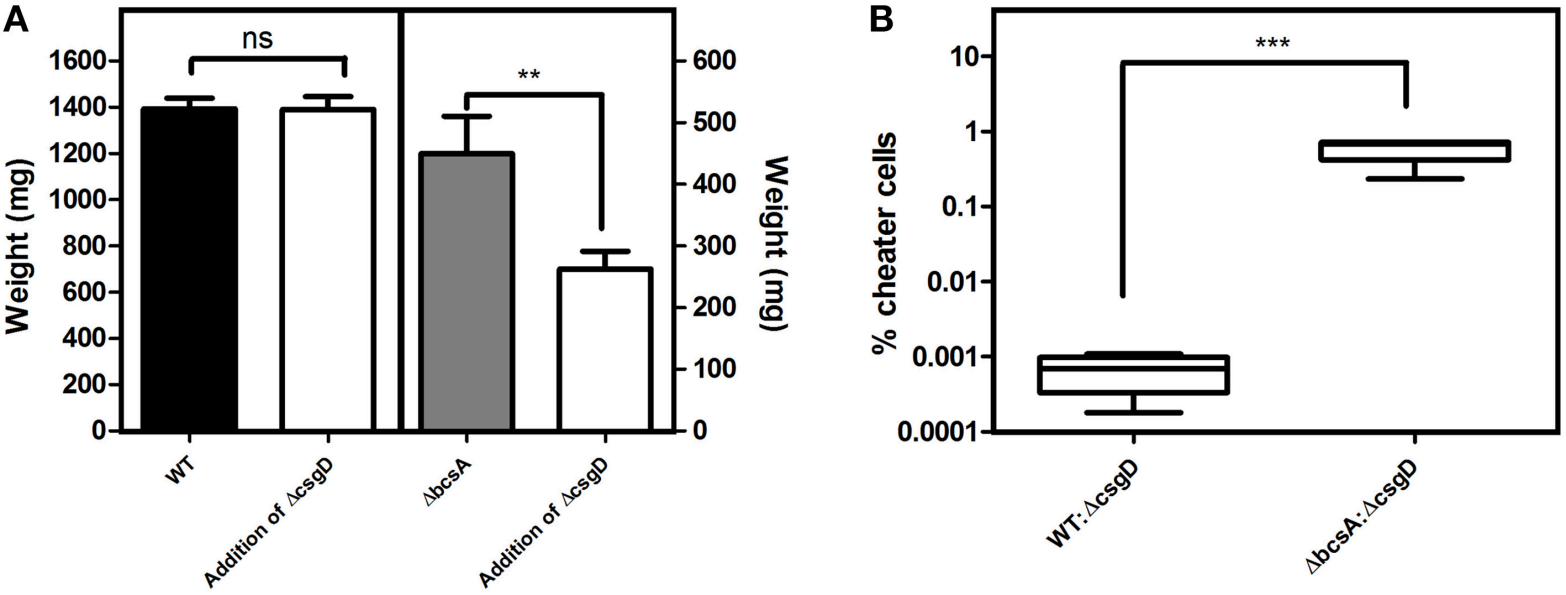
**Consequences of adding the non-producing cells post-biofilm formation**. **(A)** Productivity of the biofilm. Δ*csgD* cells were added to the 3rd day old biofilm and the dry weight was determined on 5th day post-biofilm formation. **(B)** Percentage of non-producing cells infiltrated into the biofilm on 5th day post-biofilm formation after addition of Δ*csgD* cells on the 3rd day biofilm. Error bars indicate the SEM (*n* ≥ 5). Unpaired *t*-test was used to determine significance. (^**^P < 0.01, ^***^P < 0.001).

As the cell types were spatially assorted up to 6 μm distances in the biofilm (Figure 6D), we speculated that cellulose fibers, which are up to 15 μm long, is possibly exploited by the non-producing cells to stick, unlike the curli fibrils that tightly surround the producer cells (Serra et al., 2013). Therefore, a Δ*bcsA* that does not produce cellulose but forms a fragile biofilm was co-cultured with Δ*csgD* and the resultant biofilm was spatially analyzed. It was observed that the paired cross-correlation value was above 1.0 at shorter distances (Figure 6D) suggesting that curli is exploited by the non-producing cells to stay in the pellicle, in the absence of cellulose. However, in Figure 3D, we observed that the non-producers were not able to weaken the system post-biofilm formation. We therefore speculated from the above data, that the cellulose physically prevents the non-producers toward the microcolony of producer cells (Figure 8). Further, the non-producers were added to 3 day old biofilm of WT and Δ*bcsA* pellicle, the dry weight of which was checked after incubating for 5 days. It was observed that the dry weight of the WT biofilm had not reduced while the dry weight of Δ*bcsA* biofilm decreased significantly (Unpaired *t*-test, *P* < 0.01, *n* ≥ 5) relative to their controls (Figure 9A). In addition, the viable count shows that the percentage of non-producing cells in the biofilm was significantly (Unpaired *t*-test, *P* < 0.001, *n* ≥ 5) higher in the Δ*bcsA* biofilm (Figure 9B). Altogether, these results suggest that the cellulose keeps the non-producers at bay from the biofilm forming bacterial microcolony.

## Discussion

CsgD, which activates the matrix components for the biofilm formation is influenced by oxygen tension, temperature, and other nutrients (Gerstel and Römling, 2001). The LS condition was shown to induce higher gene expression of *csgD* relative to the HS condition (Figure S1). The *csgD* expression correlated with the low fitness of producing cells in competition with the non-producers, indicating that metabolic cost is incurred for production of the EPS (Figure 1B). In the submerged biofilm, the nutrient diffuses from top (bulk phase) to down (depth of biofilm) and the cells grow toward the bulk phase, whereas in the pellicles, oxygen can be accessed from the upper part and other nutrients from the liquid below. Scher et al. (2005) proposed that the submerged biofilm formation precedes that of pellicle, and the pellicle floats by attaching to the wall. In contrast, we found initiation of pellicle formation at 24 h of incubation and the development was in parallel to the submerged biofilm (Figure 2). However, the biofilm pellicle of the *S*. Typhimurium strain we used did not sink when they were scraped at the edges with the help of tweezers, therefore the role of buoyancy cannot be ruled out. Nevertheless, the Δ*csgD* cells did not form the pellicle or a submerged biofilm, suggesting that the CsgD-dependent components are required for both the kinds of biofilm.

Introduction of non-producer cells in 1:1 ratio with WT, reduced the submerged biofilm productivity (Unpaired *t*-test, *P* = 0.039, *n* ≥ 6) (Figure 3A), but a more pronounced effect was seen on the biofilm pellicle morphology (Figure 3B and Video S1). By computational modeling, Xavier and Foster (2007) observed that the EPS producers in submerged biofilm gain more benefit than non-producers because, the EPS pushes the producing cells toward the surface, which is abundant in nutrients including oxygen. However, Popat et al. (2012) observed reduction in the overall productivity of *P. aeruginosa* submerged biofilm in presence of quorum sensing cheater cells. The biofilm pellicle, which usually comprises thick layer of biomass with wrinkles, was fragile and devoid of wrinkles after the non-producer infiltration (Figure 3B). The pellicle is an elastic polymer and EPS matrix helps form the wrinkles or deformations due to compressive stress in a confined space (Trejo et al., 2013). In addition, wrinkle formation helps the cells toward maximizing oxygen availability (Dietrich et al., 2013). However, growth of non-producer cells made the pellicle devoid of wrinkles (Figure 3B), possibly due to decrease in the ratio of matrix materials to the cell number. Productivity, in terms of dry weight of the pellicle decreased significantly (One-way ANOVA, *P* < 0.001, *n* ≥ 6) according to the ratio of WT to Δ*csgD* in the initial inoculum, suggesting that the frequency of non-producers do determine the strength of the biofilm (Figure 3C). EPS could act as a public good in the context of biofilm lifestyle, because the matrix confers several benefits to the biofilm cells (Flemming and Wingender, 2010), including higher oxygen accessibility than planktonic cells in the bottom (Rainey and Rainey, 2003). It was clearly apparent in the confocal image (Figure 6B) where the non-producing cells, which otherwise do not form biofilm, stays in the biofilm pellicle formed by the WT cells, indicating that the biofilm matrix indeed acts as a public good. As biofilm is the predominant form of lifestyle that helps the organism colonize surfaces, the emergence of non-producers could thus have important implications in colonizing surfaces by bacteria in nature.

We hypothesized that introducing non-producing cells could weaken the biofilm and sensitize it toward antibacterial compounds due to reduction in the biofilm strength and cohesion (Figure 3). Accordingly, the biofilm harboring non-producers was more sensitive to sodium hypochlorite and ciprofloxacin antibiotic than the WT biofilm (Figure 4). Increased exposure to chlorine due to increased diffusion of the antibacterials could be one possible reason making the cells more susceptible. DeBeer et al. (1994) observed poor penetration of chlorine into the submerged biofilm of *P. aeruginosa* and *Klebsiella* through microsensors. Popat et al. (2012) also observed an increased susceptibility of *P. aeruginosa* biofilm cells in the presence of cheats to tobramycin antibiotic. Diffusion took 20 min to achieve MIC in *K. pneumoniae*, and nearly 40 min for 25.32% of the initial levels of ciprofloxacin to reach the depths of *P. aeruginosa* biofilm (Anderl et al., 2000; Arabski et al., 2013). However, the fragile biofilm formed in the co-culture (Figure 3B) was found to be sensitive to the antibacterial stresses (Figures 4A–C) due to possible decrease in the matrix to cell ratio, where the antibiotic diffusion to reach the MIC concentrations would have been fast. The non-producing cells were more susceptible to the antimicrobials used in this study (Figure 4D). Cellulose was shown to be critical for the tolerance toward chlorine in *Salmonella* biofilms (Solano et al., 2002). Cellulose possibly acts as a shield to the biofilm cells by reducing the hypochlorous acid. However, the non-producers anchor to cellulose produced by the WT cells leading to their growth in the periphery of the WT microcolony (Figures 6, 8). This possibly enhanced the exposure of these cells to the antimicrobial stress than the WT, making it more vulnerable. Smukalla et al. (2008) also observed the non-flocculating cells of yeast to be more vulnerable to antimicrobial stresses. Biofilm lifestyle is predominant in around 80% of the bacterial infections, and the biofilm bacteria are usually refractive to commonly used antibacterials (Römling and Balsalobre, 2012). Therefore, novel therapeutic strategies can be developed in future by tweaking the social behavior in bacteria.

*In vivo* experiments in mice indicated that the biofilm formation is a necessary step during the initial stages of pathogenesis (Figure 5). We speculate that biofilm formation might be necessary to colonize the intestinal mucus or the epithelium before preceding the downstream pathogenic processes. Ledeboer and Jones (2005) showed biofilm formation of *S*. Typhimurium on intestinal epithelium of chicken. Biofilm formation might also possibly be an important lifestyle of survival in different organs for the chronic persistence. Latasa et al. (2005) observed a substantial decrease in the colonization efficiency of BapA mutant of *Salmonella* in different organs, while Crawford et al. (2008) showed that CsgD-dependent expression of O-antigen capsule for biofilm formation on cholesterol gallstones was enhanced by the presence of bile. Altogether, these data suggests that biofilm formation is important in various stages of pathogenesis. White et al. (2006) observed *csgD* gene expression in intestine, liver and to some extent in the MLN. The percentage of non-producers infiltration in natural biofilm could potentially influence the pathogenesis (Figure 5). As our data and other results indicate, biofilm formation could be an initial strategy of the *Salmonella* during pathogenesis. Importantly, we speculate that they may form biofilm bunkers in the intestinal mucus before they proceed to downstream infectious process. We also speculate that the infection caused by the non-producer infiltrated biofilm cells could show different antibiotic sensitivity as compared to the WT biofilm infection.

A spatial assortment of the producing and non-producing cell types were observed in the confocal images of the co-cultured biofilm (Figure 6). Moreover, the non-producers were located in the periphery of the WT microcolony (Figure 6B). Dugatkin et al. (2008) observed cheaters on the edges of the microcolony in the context of antibiotic resistant bacteria that secrete antibiotic neutralizing substances. Interestingly, the non-flocculating cells of yeast were also observed to survive at the periphery of flocculating yeast (Smukalla et al., 2008). A reciprocal attachment of the flocculating yeasts cells occurred by the help of FLO1, which is proposed to be a green beard gene and the FLO1^−^ cell types were edged out due to the failure of strong reciprocal adhesion (Smukalla et al., 2008). Latasa et al. (2005) proposed that BapA adhesin is important for recruitment of BapA expressing cells in the *Salmonella* Enteritidis biofilm, which may possibly act similarly to the FLO1 gene of the yeast cells. However, our results implicate cellulose to the observed spatial assortment (Figure 6). Dyken et al. (2013) demonstrated that the population range expansion of cooperators, which produce the public good (here invertase enzyme) supports spatial assortment of the cooperators from defectors. They observed that the cooperators were localized at the expanding frontiers of the colony. Though there was spatial patterning of the cell types, it was in contrast to what we observe in the biofilm pellicle, where the non-producers were at the periphery (Figure 6B). The above cited references show that spatial assortment of the populations occur during conflict, however, Momeni et al. (2013) demonstrated that intermixing of the population cell types occur when they cooperate. We reasoned that cellulose possibly influenced this spatial assortment of the cell types (Figure 6B), because curli was found to localize around the producer cell, while cellulose was observed to be an inter-cellular scaffold by Serra et al. (2013). Cellulose fibers could also intertwine with the flagella and reach cells up to 15 μm long in *E. coli* macrocolonies (Serra et al., 2013). The data shown by Dyken et al. (2013) and Momeni et al. (2013) display that the spatial structure (assortment or intermixing) is formed during metabolic conflict or cooperation, but our results and others (Drescher et al., 2014; Gestel et al., 2014) show implication of the EPS for the spatial structure. It can thus be conceived that conflict may induce spatial assortment, and intermixing when there is cooperation. Our results also indicate cellulose to be the main public good rather than the curli or the BapA adhesin (Figure 8). We speculate that curli proteins possibly act as club goods exclusively for the matrix producers (Figure 8), while BapA may mediate the recruitment of matrix producers (Latasa et al., 2005). However, cellulose acts as a barrier for the non-producing cells to intrude the WT microcolony, and this may possibly be one of the conflict-mediating mechanisms to thwart the non-cooperators from destabilizing the cooperative system (Figures 8, 9). Keeping the non-producers away from the microcolony may also aid the cooperators to direct other secreted public goods to its kin. For, example, Drescher et al. (2014) demonstrated that EPS is important for directing the chitinase only to its kin. However, as we earlier discussed about developing novel therapies for disrupting biofilms by tweaking the cooperative behavior of bacteria, the bottleneck in *Salmonella* system could be that of cellulose.

Biofilm development is a long-term survival strategy of *Salmonella* in nature (White et al., 2006), and it is important for *Salmonella* cells to form a persistent biofilm state inside the host (Ledeboer and Jones, 2005; Crawford et al., 2008). Biofilm also provides resilience to the bacteria against environmental stresses like the host immune system, antibiotics, heavy metals, etc., (Joseph et al., 2001; Scher et al., 2005; Kai-Larsen et al., 2010; Römling and Balsalobre, 2012). However, our results show that the infiltration of non-producers in the biofilm could have major consequences to this long-term lifestyle including the increase in vulnerability to the antibacterials and delay in pathogenesis. Cellulose can have a dual function of (a) being the main public good, where the non-producers adhere and get oxygen accessibility and, (b) to keep away the non-producers from the producer microcolony. As biofilms are a major burden in medicine and biofouling, novel strategies involving an evolutionary approach can be explored to weaken the biofilms and sensitize it toward the antimicrobials.

## Author contributions

CS and DC conceived the study; CS, ME, and DG performed the experiments; CS performed the analysis; ME and DG assisted with the data analysis; CS, DG, and DC wrote the manuscript.

### Conflict of interest statement

The authors declare that the research was conducted in the absence of any commercial or financial relationships that could be construed as a potential conflict of interest.

## Supplementary material

Table S1 **Primers used in this study**.

Figure S1 **Quantitative Real-Time PCR of the *csg*D gene expression at LS and HS conditions**. Error bars indicate Standard deviation (*n* ≥ 3).

Figure S2 **Pathogenic burden in different organs of mice**. BALB/c mice were infected with 10^7^ ml^−1^ of *S. Typhimurium* of WT and Δ*csgD* cells. Error bars indicate SEM (*n* = 5). Mann-Whitney *U* test was performed to determine the significance.

Video S1 **Consequence of non-producer infiltration in the biofilm**. The strength of WT biofilm (1st well) is higher, while the non-producer infiltrated biofilm (2nd well) is fragile and devoid of wrinkles.
