# Supplementary material for: Infiltration of Matrix-Non-producers Weakens the Salmonella Biofilm and Impairs Its Antimicrobial Tolerance and Pathogenicity
Source: Front Microbiol. 2015 Dec 23;6:1468. doi: 10.3389/fmicb.2015.01468 (PMC4688346; doi:10.3389/fmicb.2015.01468)
Supplement: Table S1 — Primers used in this study. [file Table1.DOCX]

| **Primer name/description** | **Sequence 5’ to 3’** |
| --- | --- |
| *csgD* gene inactivation Forward Primer | CTCTGCTGCTACAATCCAGGTCAGATAGCGTTTCATGGCCGTGTAGGCTGGAGCTGCTTC |
| *csgD* gene inactivation Reverse Primer | CAGCTGTCAGATGTGCGATTAAAAAAAGTGGAGTTTCATCCATATGAATATCCTCCTTAG |
| Confirmation of *csgD* gene inactivation Forward Primer | CAATCCGGGAACCTCGACTTC |
| Confirmation of *csgD* gene inactivation Reverse Primer | ATTTCAACCCACAGCAGTGC |
| *bcsA* gene inactivation Forward Primer | CGCACACATCCAGGACAATTTTCTTTTCATCGCATTATCAGTGTAGGCTGGAGCTGCTTC |
| *bcsA* gene inactivation Reverse Primer | TGCATGATGCGGGCGACAAAACGTCCGCCGGGAGCCTGCGCATATGAATATCCTCCTTAG |
| Confirmation of *bcsA* gene inactivation Forward Primer | CGTCATGAAAGCAGGAAACG |
| Confirmation of *bcsA* gene inactivation Reverse Primer | TCGTTAGCCGCCGAAGAGGT |
| Forward Primer for qPCR | ACGATTATCCCTACCGTGAA |
| Reverse Primer for qPCR | GCGGACTCGGTGCTGTTGTA |
