# Supplementary figures and images for: Infiltration of Matrix-Non-producers Weakens the Salmonella Biofilm and Impairs Its Antimicrobial Tolerance and Pathogenicity
Source: Front Microbiol. 2015 Dec 23;6:1468. doi: 10.3389/fmicb.2015.01468 (PMC4688346; doi:10.3389/fmicb.2015.01468)

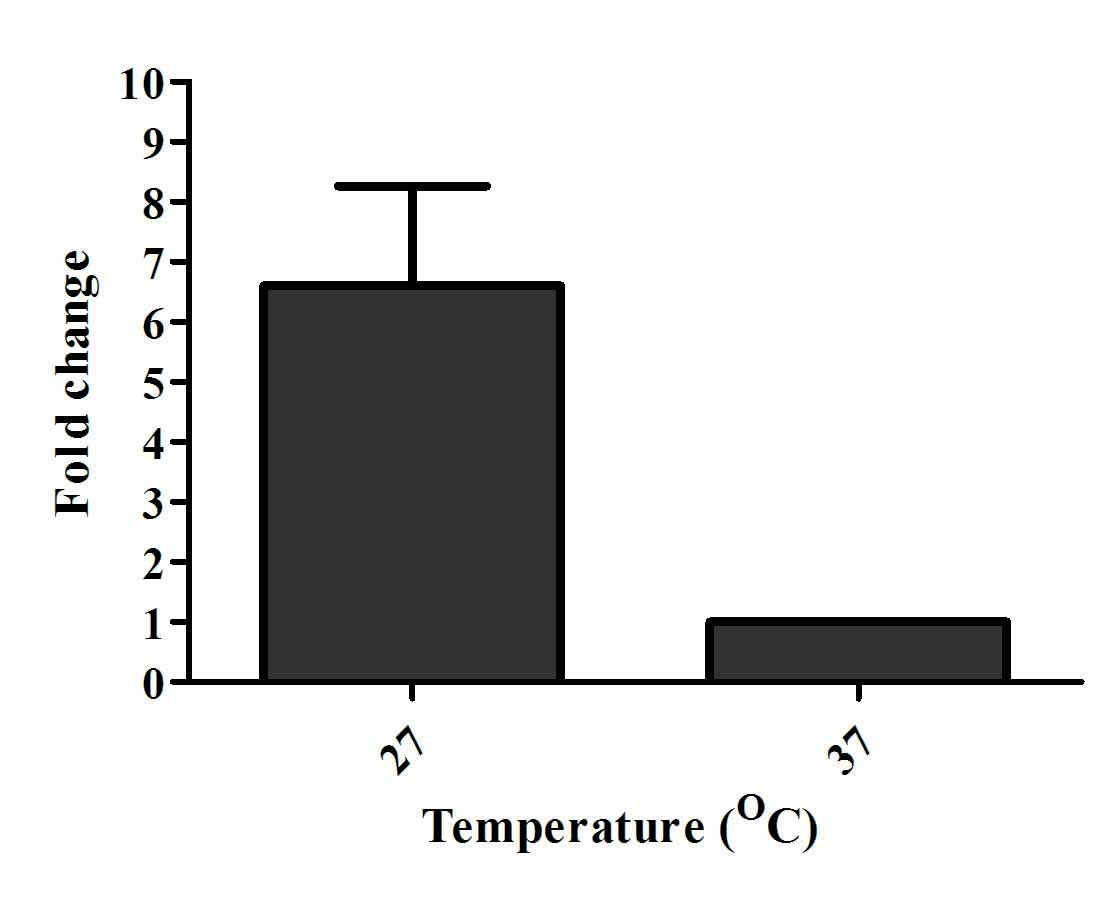

Supplement: Figure S1 — Quantitative Real-Time PCR of the csgD gene expression at LS and HS conditions. Error bars indicate Standard deviation (n ≥ 3). [file Image1.JPEG]

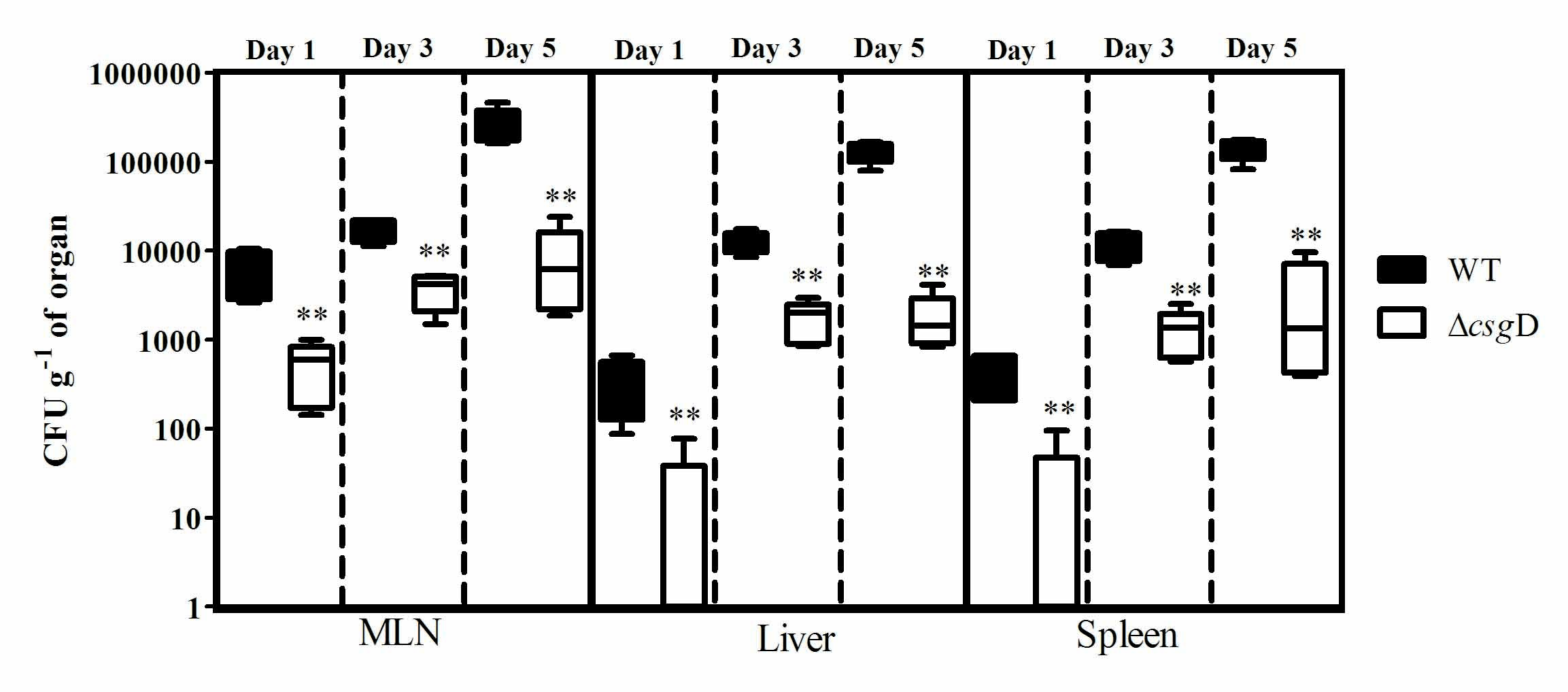

Supplement: Figure S2 — Pathogenic burden in different organs of mice. BALB/c mice were infected with 107 ml−1 of S. Typhimurium of WT and ΔcsgD cells. Error bars indicate SEM (n = 5). Mann-Whitney U test was performed to determine the significance. [file Image2.JPEG]
